# Critical Functions of Rpa3/Ssb3 in S-Phase DNA Damage Responses in Fission Yeast

**DOI:** 10.1371/journal.pgen.1001138

**Published:** 2010-09-23

**Authors:** Santiago Cavero, Oliver Limbo, Paul Russell

**Affiliations:** Department of Molecular Biology, The Scripps Research Institute, La Jolla, California, United States of America; National Cancer Institute, United States of America

## Abstract

Replication Protein A (RPA) is a heterotrimeric, single-stranded DNA (ssDNA)–binding complex required for DNA replication and repair, homologous recombination, DNA damage checkpoint signaling, and telomere maintenance. Whilst the larger RPA subunits, Rpa1 and Rpa2, have essential interactions with ssDNA, the molecular functions of the smallest subunit Rpa3 are unknown. Here, we investigate the Rpa3 ortholog Ssb3 in *Schizosaccharomyces pombe* and find that it is dispensable for cell viability, checkpoint signaling, RPA foci formation, and meiosis. However, increased spontaneous Rad11^Rpa1^ and Rad22^Rad52^ nuclear foci in *ssb3Δ* cells indicate genome maintenance defects. Moreover, Ssb3 is required for resistance to genotoxins that disrupt DNA replication. Genetic interaction studies indicate that Ssb3 has a close functional relationship with the Mms1-Mms22 protein complex, which is required for survival after DNA damage in S-phase, and with the mitotic functions of Mus81-Eme1 Holliday junction resolvase that is required for recovery from replication fork collapse. From these studies we propose that Ssb3 plays a critical role in mediating RPA functions that are required for repair or tolerance of DNA lesions in S-phase. Rpa3 orthologs in humans and other species may have a similar function.

## Author Summary

Proteins that bind single-stranded DNA (ssDNA) are essential for DNA replication, most types of DNA repair including homologous recombination, DNA damage signaling, and maintenance of telomeres. In eukaryotes, the most ubiquitous and abundant ssDNA binding protein is Replication Protein A (RPA), a 3-subunit protein complex consisting of large (Rpa1), medium (Rpa2), and small (Rpa3) subunits. Rpa1 and Rpa2 directly bind ssDNA, whilst the function of Rpa3 is largely unknown. Here, we discover that in fission yeast a 2-subunit complex of Rpa1 and Rpa2 is sufficient for the essential DNA replication function of RPA and its role in homologous recombination repair of double-strand breaks. Rpa3 is not required for these functions, but it is needed for survival of many types of DNA damage that stall or collapse replication forks. Genetic studies indicate close functional links between the Rpa3-dependent activities of RPA, the repair of collapsed replication forks by Mus81-Eme1 Holliday junction resolvase, and the newly discovered Mms1-Mms22 protein complex that is essential for resistance to genotoxins that disrupt DNA replication.

## Introduction

Preserving genome integrity in eukaryotic organisms depends on integrated mechanisms of DNA replication, DNA repair and telomere maintenance, which are all overseen by checkpoint control systems. Most genome maintenance proteins target specific types of DNA lesions, but a few have much more generalized functions. Of the latter class, perhaps the best-known example is replication protein A (RPA). Also known as single-stranded DNA-binding protein (SSB) or replication factor A (RFA), RPA consists of Rpa1 (∼70 kDa), Rpa2 (∼36 kDa) and Rpa3 (∼14 kDa), that together comprise the major single-stranded DNA (ssDNA) binding activity in eukaryotic cells [1]–[4]. RPA was originally described as a factor that is essential for replication initiation and elongation of SV40 virus DNA in cell extracts [5]–[7], and has since been shown to be required for nucleotide excision repair (NER) and mismatch repair (MMR) in vitro [8], [9]. It also stimulates the activity of homologous recombination (HR) repair proteins in vitro. Indeed, RPA is thought to be a critical factor in every DNA replication or repair process that involves ssDNA [1], [3].

All the known cellular functions of RPA depend on its ability to bind ssDNA [1], [3], [10], [11]. Although a complete 3-dimensional structure of RPA is lacking, structural and biochemical analyses have provided a detailed picture of its domain organization. In essence, RPA is made of 6 oligosaccharide/oligonucleotide binding (OB)-folds: 4 in Rpa1, and 1 each in Rpa2 and Rpa3. Direct binding to ssDNA is mediated by the OB-folds in Rpa1 and Rpa2. The OB-fold in Rpa3 is thought to mediate protein interactions that are required to stabilize the RPA heterotrimer [12], [13].

Teasing apart the *in vivo* functions of RPA subunits has been a challenging task because RPA is essential for cell viability. Almost all of this work has been carried out with the budding yeast *Saccharomyces cerevisiae*, where gene disruption studies established that all 3 subunits are required for cell viability [14]. Most genetic studies have been carried out with Rfa1, the ∼70kDA subunit, in which analyses of temperature sensitive or hypomorphic mutants have uncovered defects in DNA replication, recombination, repair, telomere maintenance, and DNA damage checkpoint signaling [3], [4]. Participation in checkpoint signaling has been traced to an interaction with Mec1-Ddc2 checkpoint kinase, which is orthologous to ATR/ATRIP in mammals and Rad3/Rad26 in the fission yeast *Schizosaccharomyces pombe* [15], [16]. Studies of Rfa2 mutants in budding yeast demonstrate its importance for DNA replication, recombination, repair, and telomere maintenance, although a checkpoint-signaling defect has yet to be established [1], [3]. In contrast to Rfa1 and Rfa2, very little is known about the function of Rfa3 *in vivo*, with the analyses limited to an N-terminal truncation mutant and a temperature sensitive allele [17].

We have been using fission yeast to investigate the cellular responses to DNA damage in S-phase. Many of these studies have focused on the effects of camptothecin (CPT), which is the prototype of a class of anticancer drugs that stabilize covalent DNA-topoisomerase I complexes by preventing the religation step of topoisomerase I [18]. When a replication fork encounters the CPT-Topoisomerase I complex, it can break, either through direct collision with the CPT-Topoisomerase I complex, or through formation of positive supercoils that stall the fork and can lead to its collapse [19]. In either case, the resulting DNA damage is a one-ended DSB that is subsequently repaired by a homologous recombination repair pathway that creates a Holliday junction in the process of reestablishing the replication fork [20]. Notably, the Mus81-Eme1 Holliday junction resolvase is essential for CPT resistance but plays no role in survival of DSBs created by ionizing radiation (IR), which are repaired primarily by a synthesis-dependent strand annealing (SDSA) mechanism that does not require resolution of Holliday junctions [21]–[23]. We also recently described the Mms1-Mms22/Mus7 protein complex in fission yeast, which like its counterpart in budding yeast, appears to play a very important but as yet poorly understood role in the survival of genotoxins that interfere with DNA replication [24]–[28].

In an effort to more fully characterize the response of fission yeast to replication-associated DNA damage, we carried out a genome-wide screen to identify genes required for CPT resistance. Using a haploid deletion library, we identified a group of CPT sensitive mutants, amongst the most sensitive were mutants for *mms22* or *ssb3*, the latter of which encodes Rpa3. In this report we characterize Ssb3 and explore its role in recovery from DNA damage in S-phase.

## Results

### Rpa3/Ssb3 is dispensable for cell viability in fission yeast

We screened an *S. pombe* haploid deletion library to identify genes required for CPT resistance. In agreement with another recent study [29], we found that *mms22Δ* and *ssb3Δ* mutants were amongst the most CPT-sensitive strains in the library (see below). Identification of *mms22Δ* was anticipated from other studies [24]–[27]. However, as the three subunits of RPA are essential for cell viability in *S. cerevisiae*, and at least the large subunit Rad11^Ssb1/Rpa1^ is essential in *S. pombe* [30], it was unexpected that an *ssb3Δ* mutant should be viable in *S. pombe*.

**Figure 1:**
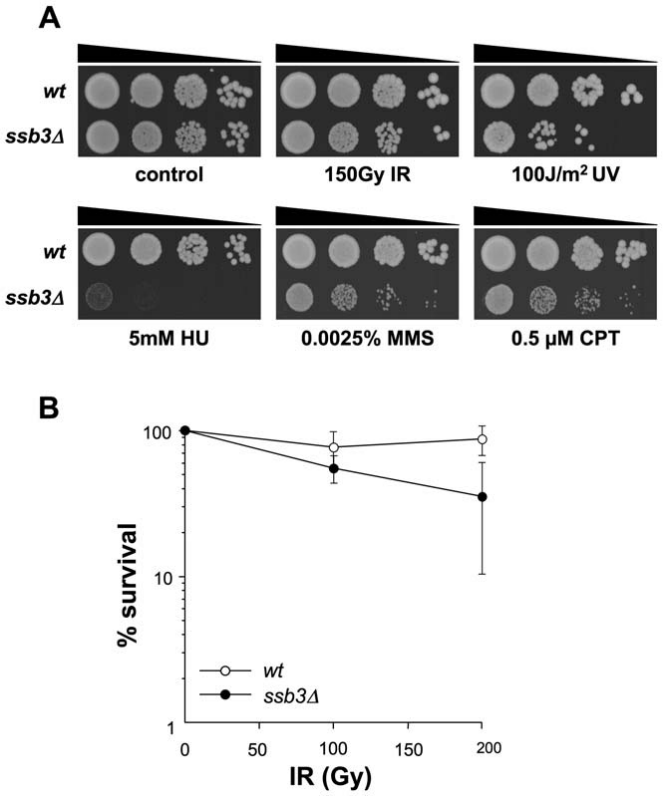
Genotoxin sensitive phenotypes of *ssb3Δ* mutants. (**A**) *ssb3Δ* cells are sensitive to a number of DNA-damaging agents, particularly those that disrupt DNA replication. Tenfold serial dilutions of cells were plated on YES agar medium, exposed to the indicated DNA-damaging agents and incubated at 30°C for 3–4 days. (**B**) Elimination of Ssb3 causes a weak IR-sensitive phenotype. Mean values of three different experiments are shown, with error bars representing the standard deviation of the mean.

As some alleles in the Bioneer *S. pombe* deletion library are incomplete deletions, and errors can arise when screening arrayed mutant libraries, it was important to characterize the structure of the presumptive *ssb3::KanMX4* mutant in the library. This analysis revealed that the *ssb3::KanMX4* mutant was correctly arrayed in the library but the deletion was incomplete. The Bioneer *ssb3::KanMX4* allele can potentially encode a protein having the first 21 amino acids of Ssb3 (S. Cavero and P. Russell, unpublished data). Mindful that the C-terminal 52 amino acids of *S. cerevisiae* Rfa3 are sufficient for function *in vivo* [17], we designed a new *ssb3::KanMX6* construct that completely eliminates the *ssb3* ^+^ open reading frame (see Materials and Methods). Haploid cells harboring this allele were viable and sensitive to CPT, confirming that Ssb3 is not required for cell viability in fission yeast but is required for CPT resistance (Figure 1A).

### Ssb3 is required for survival of DNA damage in S-phase

We next used serial dilution assays to assess the sensitivity of *ssb3Δ* cells to a range of genotoxins (Figure 1A). These studies confirmed that *ssb3Δ* cells are very sensitive to CPT. These cells were also sensitive to a low dose (0.0025%) of methyl methanesulfonate (MMS), which interferes with replication fork progression by alkylating DNA, and to hydroxyrurea (HU), which inhibits DNA replication by poisoning ribonucleotide reductase (Figure 1A). The *ssb3Δ* cells were also sensitive to UV, which creates cyclobutane dimers and other lesions that impede replication forks. Interestingly, *ssb3Δ* cells were only modestly sensitive to ionizing radiation (IR), the primary toxic effects of which are DSBs that are repaired in G2 phase (Figure 1A,B). These data show that Ssb3 is required for resistance to a range of genotoxins, particularly those that interfere with DNA replication.

### Ssb3 is not required for meiosis

**Figure 2:**
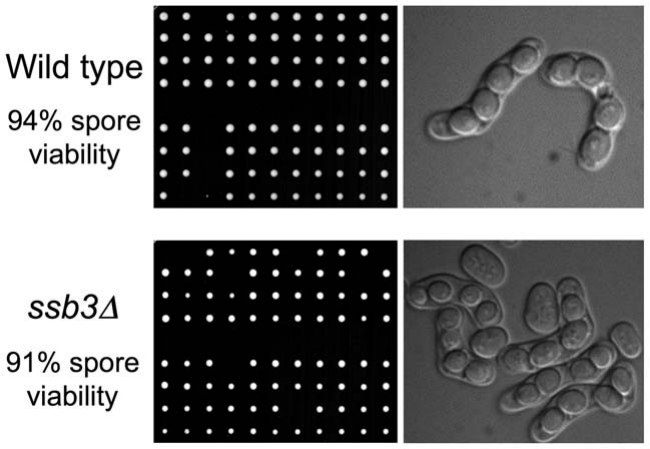
Ssb3 is not required for meiosis. Wild type x wild type or *ssb3Δ*×*ssb3Δ* matings were analyzed by tetrad dissection (left panel). Four-spore asci appeared normal in both matings (right panel).

The weak IR sensitivity of *ssb3Δ* cells suggested that Ssb3 is largely dispensable for homologous recombination-mediated repair of DSBs in mitotic cells. To confirm and extend these findings we analyzed meiosis, in which homologous recombination repair and crossover resolution of programmed meiotic DSBs is required for proper chromosome segregation and spore viability [31]. Tetrad analysis of an *ssb3Δ*×*ssb3Δ* cross yielded 91% spore viability, which was only a slight decrease from the 94% spore viability of a *ssb3* ^+^×*ssb3* ^+^ cross (Figure 2). Consistent with this high spore viability, microscopic observation showed that the asci from the *ssb3Δ*×*ssb3Δ* cross were indistinguishable from wild type. From these results we conclude that Ssb3 is not required for meiotic DSB repair.

### DNA damage checkpoint signaling is intact in *ssb3Δ* cells

**Figure 3:**
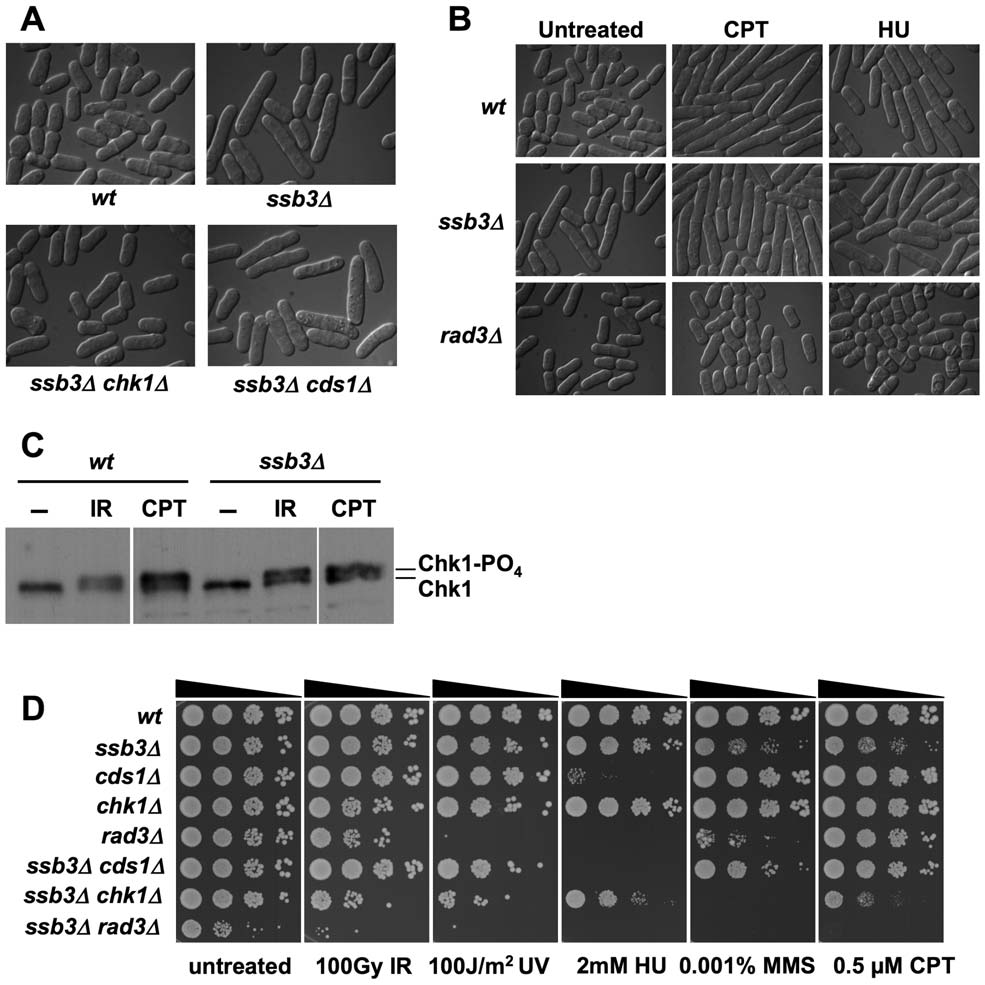
DNA damage checkpoint responses in *ssb3Δ* cells. (**A**) *ssb3Δ* cells are elongated even in the absence of DNA damaging agents. Elimination of Chk1 suppresses this elongated phenotype. The mean relative cell lengths of mutants (normalized to wild type = 1.00±0.03 SD) are: *ssb3Δ* = 1.21±0.04; *ssb3Δ chk1Δ* = 1.01±0.01; *ssb3Δ* cds1*Δ* = 1.21±0.04. Data are derived from 3 independent measurements of 100 cells each (**B**) *ssb3Δ* cells arrest division and elongate in response to CPT, showing that the DNA damage checkpoint is intact. The *rad3Δ* strain is a checkpoint defective control. (**C**) Chk1 undergoes activating phosphorylation in *ssb3Δ* cells. After CPT or IR treatment, Chk1 is phosphorylated in *ssb3Δ* cells as well as in control cells, as indicated by the appearance of a slow-mobility species. Samples were processed for immunoblot analysis of HA-tagged Chk1. (**D**) Phenotypes of *ssb3Δ* cells in combination with checkpoint kinase disruptions. Tenfold serial dilutions of cells were plated on YES agar medium, exposed to the indicated DNA-damaging agents, and incubated at 30°C for 3–4 days.

Whilst *ssb3Δ* cells are viable and have only a slightly reduced growth rate, they are elongated relative to wild type (Figure 3A). As this phenotype typically results from activation of a cell cycle checkpoint, we crossed *ssb3Δ* into strains lacking the Cds1^Rad53/Chk2^ DNA replication checkpoint kinase or the Chk1 DNA damage checkpoint kinase [32]. Microscopic analysis revealed that the *ssb3Δ* elongated cell morphology phenotype required Chk1 but not Cds1 (Figure 3A). Various types of DNA damage activate Chk1, whereas Cds1 is primarily activated in response to stalled replication forks. Therefore, these findings indicate that the elongated morphology of *ssb3Δ* cells is caused by spontaneous DNA damage activating a DNA damage checkpoint that delays the onset of mitosis.

The suppression of the *ssb3Δ* elongated cell morphology phenotype by *chk1Δ* suggested that the DNA damage checkpoint is intact in the absence of Ssb3. To further test this hypothesis, we observed the response of *ssb3Δ* cells to CPT or HU treatment. Both wild type and *ssb3Δ* cells underwent cell cycle arrest in response to these treatments, whereas cells lacking Rad3^Mec1/ATR^ failed to arrest division (Figure 3B). Consistent with these findings, we observed that Chk1 became hyper-phosphorylated in response to IR or CPT treatment (Figure 3C), which is indicative of an intact DNA damage checkpoint response [33].

These data showed that Ssb3 is not required for the Rad3-Chk1 branch of the DNA damage checkpoint pathway. To confirm this proposition, we carried out genetic epistasis studies with *rad3Δ*, *chk1Δ* and *cds1Δ* mutations. By crossing *rad3Δ* and *ssb3Δ* mutants, we discovered a strong genetic interaction between the two mutations, with the double mutant growing much slower than either single mutant (untreated panel in Figure 3D). Although the *chk1*Δ *ssb3Δ* double mutant did not have a strong growth defect in the absence of genotoxins, there were obvious synergistic interactions in the presence of IR, UV, HU, MMS and CPT (Figure 3D). The interactions between *cds1Δ* and *ssb3Δ* were more complicated: we observed no interactions in response to IR or UV, an additive effect in HU, and suppression in the presence of MMS or CPT (Figure 3D).

### Increased spontaneous DNA damage in *ssb3Δ* cells

**Figure 4:**
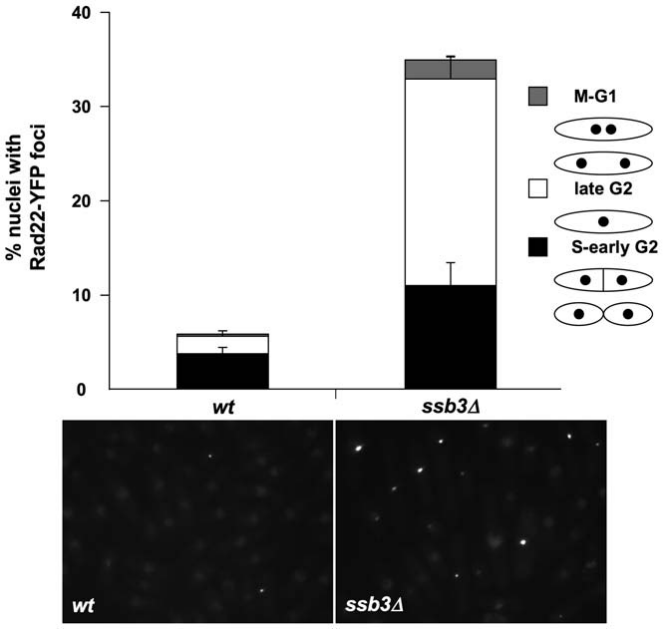
Increased Rad22 DNA repair foci in *ssb3Δ* cells. Rad22-YFP foci formation was significantly increased in *ssb3Δ* cells, especially during the S- and G2-phases of the cell cycle. Control and *ssb3Δ* cells were cultured in EMM liquid medium at 30°C until mid-log phase, photographed, and the number of nuclei with at least one Rad22-YFP foci was scored. Mean values of three different experiments are shown, with error bars representing the standard deviation of the mean.

The Chk1-dependent cell elongation in *ssb3Δ* cells suggested that they suffer spontaneous DNA damage. To explore this idea further, we monitored Rad22-YFP foci in *ssb3Δ* cells. Rad22 is the fission yeast ortholog of Rad52, which is essential for homologous recombination (HR) repair, and many mutants that have genome maintenance defects have increased numbers of Rad22 foci [34]–[36]. We observed a large increase in cells with Rad22-YFP foci in the *ssb3Δ* strain (∼34%) compared to wild type (∼6%) (Figure 4). We consistently observed that a large fraction of the Rad22-YFP foci in *ssb3Δ* cells were brighter than in wild type, indicating more extensive recruitment of Rad22. Approximately 33% of the cells with Rad22-YFP foci were septated or attached, which correlates with S-phase in fission yeast, whilst most of the other cells with Rad22-YFP foci appeared to be in G2 phase (Figure 4). These findings suggest that *ssb3Δ* cells suffer increased rates of DNA damage in S-phase.

### Ssb3 colocalizes with Rad22

**Figure 5:**
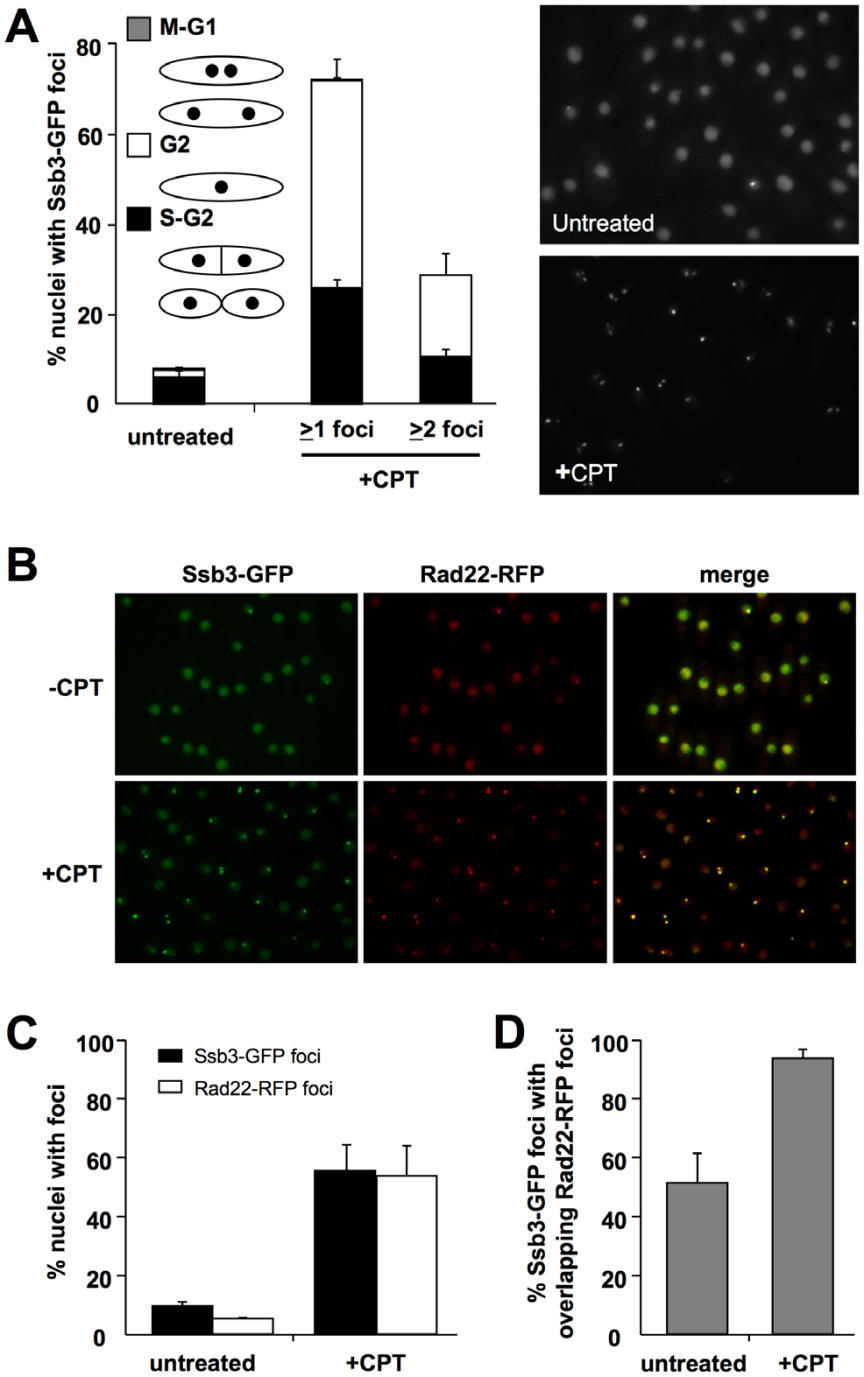
Ssb3 localizes to sites of DSBs. (**A**) Ssb3 forms nuclear foci that increase in number following DNA damage. Cells expressing endogenous Ssb3-GFP were cultured in YES liquid medium at 30°C until mid-log phase and then treated with CPT or left untreated. Nuclei with at least one or with two or more Ssb3-GFP foci were scored in three independent experiments and the mean values are represented. Error bars correspond to standard deviations of the mean. (**B**) Ssb3 colocalizes with Rad22. The majority of both spontaneous and CPT-caused Ssb3-GFP foci colocalize with Rad22-RFP foci. Cells expressing endogenous Ssb3-GFP and Rad22-RFP were cultured and CPT-treated as indicated above. Representative images are shown. (**C**) Quantification of the percentages of nuclei with Ssb3-GFP or Rad22-RFP, with or without CPT treatment. (**D**) Quantification of the percentages of Ssb3-GFP foci with overlapping Rad22-RFP foci, with or without CPT treatment. In each case, nuclei with foci were scored in three independent experiments and the mean values are plotted, with error bars corresponding to the standard deviation of the mean.

To address whether Ssb3 relocalizes to sites of DNA damage, we created a strain in which genomic *ssb3* ^+^ was modified to encode Ssb3-GFP. This strain was not noticeably sensitive to CPT, indicating that Ssb3 function is undisturbed by the C-terminal GFP fusion (S. Cavero and P. Russell, unpublished data). By live cell analysis we observed that Ssb3-GFP was exclusively nuclear, with ∼7.5% of the cells in an asynchronous population having a bright nuclear focus. This pattern is typical of HR repair proteins such as Rad22 [36]. Upon treatment with CPT, there was a large increase in the number of cells with one or more Ssb3-GFP foci (Figure 5A). Again, this is typical of HR proteins, indicating that Ssb3 is a subunit of an RPA complex involved in homologous repair of DSBs.

To confirm whether Ssb3-GFP localizes at sites of ongoing DSB repair, we created a strain that co-expresses Ssb3-GFP and Rad22-RFP from their genomic loci. In the absence of genotoxic stress, ∼50% of the Ssb3-GFP foci co-localize with Rad22-RFP foci (Figure 5B–D). However, following CPT treatment, there was nearly complete overlap of the Ssb3-GFP and Rad22-RFP foci. This result indicates that Ssb3-GFP foci represent sites of actual DSBs and support a direct role for this protein in the repair of broken replication forks. It should be noted that Ssb3-GFP has a higher nuclear fluorescence, with brighter foci than Rad22-RFP, especially in the absence of DNA damaging agents. This fluorescence difference may cause an underestimation of Rad22-RFP foci, especially in the absence of genotoxins, and hence an underestimation of colocalization of Ssb3-GFP and Rad22-RFP foci.

### Genetic interactions involving Ssb3 and Rad11

**Figure 6:**
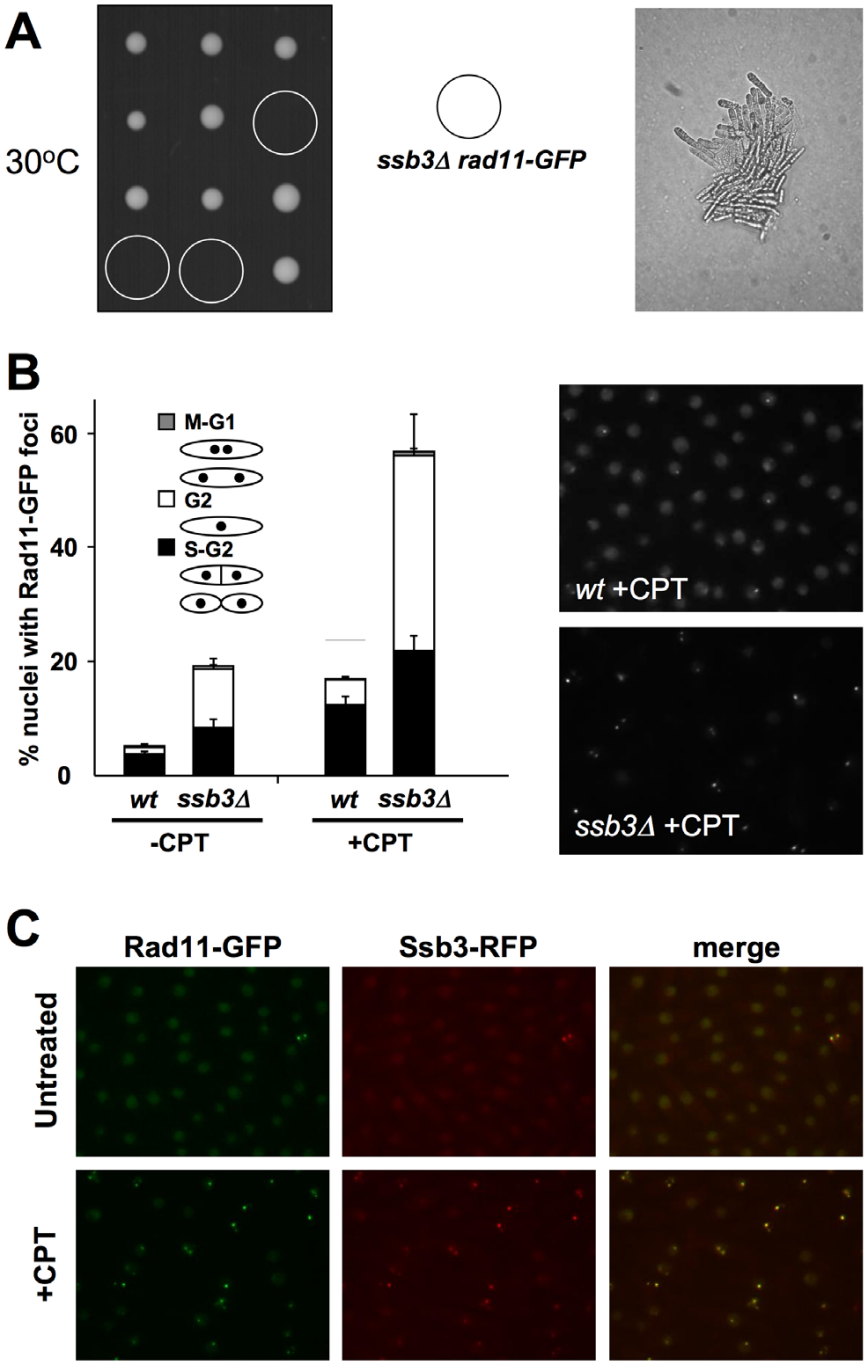
Rad11^Rpa1^ foci are increased in *ssb3Δ* cells. (**A**) *ssb3Δ rad11-GFP* show strong genetic interactions at 30°C. The left panel shows tetrad analysis of a *ssb3Δ*×*rad11-GFP* mating germinated at 30°C. The right panel shows a photomicrograph of *ssb3Δ rad11-GFP* cells from the tetrad dissection plate. (**B**) Rad11-GFP forms nuclear foci that increase in number following DNA damage even in the absence of Ssb3. Cells expressing endogenous Rad11-GFP in a wild type or *ssb3Δ* background were cultured in YES liquid medium at 25°C until mid-log phase and then treated with CPT or left untreated. Percentages of nuclei with at least one Rad11-GFP focus are shown. Quantification of Rad11-GFP foci in different cell cycle stages was determined. Foci were scored in three independent experiments and the mean values are represented. Error bars correspond to standard deviations of the mean. (**C**) Co-localization of Rad11-GFP and Ssb3-RFP foci upon treatment with CPT.

To further investigate the role of Ssb3 in RPA function, we performed a genetic cross to express GFP-tagged Rad11^RPA1^ in *ssb3Δ* cells. Although the *rad11-GFP* and *ssb3Δ* parents grew well at the standard growth temperature of 30°C, tetrad analysis uncovered a strong synthetic lethal interaction between the two alleles at 30°C (Figure 6A). Microscopic analyses performed revealed that the *rad11-GFP ssb3Δ* spores formed microcolonies of very elongated cells at 30°C (Figure 6A). A similar effect was observed with HA-tagged *rad11* (S. Cavero and P. Russell, unpublished data). These data indicated that the tags slightly impair Rad11 function or destabilize the RPA complex, making it highly dependent on Ssb3. In support of this idea, we found that spore germination at 25°C yielded healthier *rad11-GFP ssb3Δ* cells that were suitable for localization studies. As expected, the growth of these cells was temperature sensitive (S. Cavero and P. Russell, unpublished data).

We carried out live-cell analysis of Rad11-GFP localization, in the absence or presence of CPT, in wild type (*wt*) or *ssb3Δ* cells grown at 25°C. About 5% of wild type cells had Rad11-GFP nuclear foci in the absence of DNA damage (Figure 6B). This number increased ∼3.5-fold when cells were incubated with 30µM CPT for 4 hours, indicating that Rad11-containing RPA complex localizes to DSBs, as expected. Interestingly, the percentage of nuclei with Rad11-GFP foci in the absence of DNA damage was greater in *ssb3Δ* cells, probably resulting from increased spontaneous DNA damage. Similarly, after CPT treatment, the increase in Rad11-GFP foci formation was also greater in *ssb3Δ* cells compared to wild type (Fig. 6B). Therefore, the relocalization of RPA complex to DSBs does not depend on Ssb3, and instead it appears to be enhanced.

It should be noted that the frequency of Rad11-GFP foci observed in wild type cells in this experiment was less than seen for Ssb3-GFP in Figure 5. This difference most likely arises from the effect of temperature; indeed, the frequency of Ssb3-GFP foci is reduced at 25°C versus 30°C (S. Cavero and P. Russell, unpublished data). Moreover, co-expression of Rad11-GFP and Ssb3-RFP revealed almost 100% overlap of nuclear foci (Figure 6C).

### Genetic epistasis studies with *ssb3Δ*

**Figure 7:**
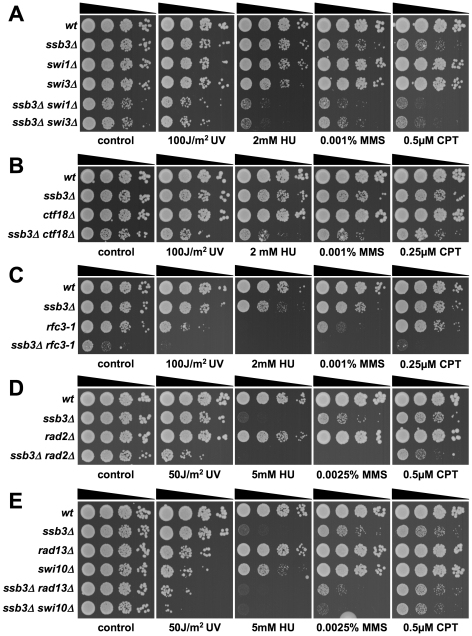
Genetic interactions involving Ssb3 and components of replication fork protection complexes, Okazaki fragment processing, and NER. Tenfold serial dilutions of cells were exposed to the indicated DNA-damaging agents and plates were incubated at 30°C for 3–4 days, except for *rfc3-1* strains which were incubated at 25°C. Representative images of repeat experiments are shown.

**Figure 8:**
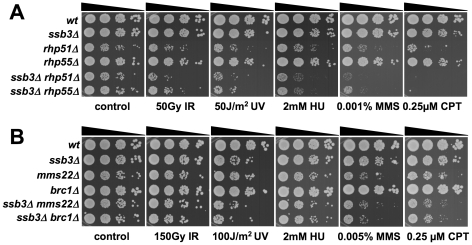
Genetic interactions between Ssb3 and Rhp51, Rhp55, Mms22, and Brc1. Tenfold serial dilutions of cells were exposed to the indicated DNA-damaging agents and plates were incubated at 30°C for 3–4 days. Representative images of repeat experiments are shown.

Having found that Ssb3 is required for survival of S-phase DNA damage and that it colocalizes with Rad22 and RPA at CPT-induced DNA damage sites, we lastly performed a genetic epistasis analysis of *ssb3Δ* with genes involved in different pathways of DNA replication or repair. Double mutant strains were created by mating and assessed for growth in dilution series on rich growth medium in the absence or presence of various genotoxins (Figures 7 and 8). The data are summarized in Table 1. In the absence of genotoxins, we detected negative genetic interactions with the Swi1^Tof1^-Swi3^Csm3^ replication fork protection complex (FPC), which is required for stable fork pausing and efficient activation of Cds1 [34], [37]; Ctf18, which is a subunit of an alternative Replication Factor C (RFC) complex that is involved in fork stabilization [38]; Rfc3, a subunit of RFC [39], [40]; Rad2, the 5′ flap endonuclease required for Okazaki fragment processing during DNA replication; Rhp55^Rad55^, a Rhp51^Rad51^ paralog that forms a heterodimer with Rhp57^Rad57^ that mediates the formation and/or stabilization of the Rad51-DNA filament required for HR repair [40]; and Brc1^Rtt107^, a 6-BRCT domain protein that is involved in survival after DNA damage in S-phase and binds phospho-histone H2A (γH2A) at sites of DNA damage [41], [42]. Under the same conditions we detected only weak or no negative genetic interactions with Rad13 and Swi10, which are required for nucleotide excision repair (NER); Rhp51, which is the Rad51 ortholog required for most types of HR repair; or with Mms22, which is required for recovery after DNA damage in S-phase [24], [25]. In the presence of genotoxins, additional negative genetic interactions with *ssb3Δ* were revealed for Rad13, Swi10 and Rhp51. Mms22 did not show a genetic interaction with Ssb3 in the presence of genotoxins, with the exception of a weak interaction in cells treated with UV (Figures 7 and 8).

**Table 1:** Summary of genetic interactions involving *ssb3Δ*.

Allele	Ortholog; function	Untreated	IR	UV	HU	MMS	CPT
*swi1Δ*	Tof1; Replication fork pausing and protection complex	Yes	n.t.	Yes	Yes	Yes	Yes
*swi3Δ*	Csm3; Replication fork pausing and protection complex	Yes	n.t.	Yes	Yes	Yes	Yes
*ctf18Δ*	Ctf18; alternative RFC subunit	Yes	n.t.	Yes	Yes	Yes	Yes
*rfc3-1*	DNA replication factor C (RFC) subunit	**YES**	n.t.	**YES**	n.t.	**YES**	**YES**
*rad13Δ*	XPG/Rad2; NER endonuclease	No	n.t.	Yes	No	Yes	No
*swi10Δ*	ERCC1/Rad10; NER endonuclease	No	n.t.	Yes	No	Yes	Yes
*rad2Δ*	Fen1/Rad27; 5′ flap endonuclease; Okazaki fragment processing	Yes	n.t.	Yes	Yes	Yes	Yes
*rhp51Δ*	Rad51; HR repair of DSBs	No	Yes	Yes	Yes	Yes	Yes
*rhp55Δ*	Rad55; Subunit of Rhp51 mediator	Yes	**YES**	**YES**	**YES**	**YES**	**YES**
*swi5Δ*	HR repair of DSBs; Subunit of alternative Rhp51 mediator	No	n.t.	Yes	Yes	Yes	Yes
*sfr1Δ*	HR repair of DSBs; Subunit of alternative Rhp51 mediator	No	n.t.	Yes	Yes	Yes	Yes
*mms1Δ*	Mms1; DNA repair in S-phase	No	No	Yes?	No	No	No
*mms22Δ*	Mms22; DNA repair in S-phase	No	No	Yes?	No	No	No
*brc1Δ*	Rtt107; DNA repair in S-phase	Yes	Yes	Yes	Yes	**YES**	**YES**
*mus81Δ*	Mus81; Holliday junction resolvase	No	No	No	No	No	No
*chk1Δ*	Chk1; DNA damage checkpoint kinase	No	Yes	Yes	Yes	Yes	Yes
*cds1Δ*	Rad53/Chk2; DNA replication checkpoint kinase	No	No	No	Yes	**P.I.**	**P.I.**
*rad3Δ*	Mec1/ATR: Checkpoint kinase that activates Chk1 and Cds1	**YES**	Yes	Yes	n.t.	Yes	Yes

Double mutants were assessed for growth in the absence or in the presence of the specified genotoxins. YES, strong negative interaction; Yes, negative interaction; No, no genetic interaction; P.I., positive interaction; n.t., not tested.

**Figure 9:**
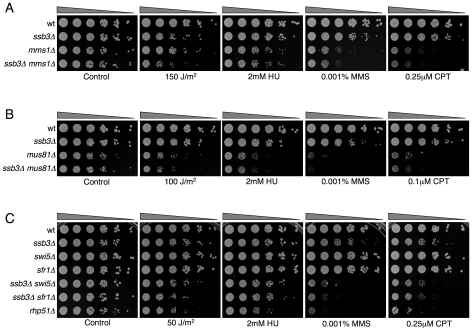
Genetic interactions between Ssb3 and Mms1, Mus81, Swi5, and Sfr1. Tenfold serial dilutions of cells were exposed to the indicated DNA-damaging agents and plates were incubated at 30°C for 3–4 days. Representative images of repeat experiments are shown.

Mms22 forms a protein complex with Mms1, and we have found that *mms1Δ mms22Δ* double mutants behave identically to either single mutant [28]. From these genetic relationships we predicted that *ssb3Δ* and *mms1Δ* should have an epistatic genetic interaction in all conditions except for UV, as was seen for *ssb3Δ* and *mms22Δ* (Figure 8). Genetic studies confirmed this prediction (Figure 9A).

Mutations that inactivate Mms1 and Mms22 have negative genetic interactions with deletions of many genome maintenance genes. One exception is Mus81, which together with Eme1 forms a Holliday junction resolvase that is required for recovery from collapsed replication forks and resolution of crossovers in meiosis [21]–[23], [43]. Having found that *ssb3Δ* has an epistatic relationship with *mms1Δ* and *mms22Δ*, we explored the genetic interactions involving *ssb3Δ* and *mus81Δ*. In the absence of genotoxins, *mus81Δ* cells grew slowly compared to wild type, but this defect was not enhanced by eliminating Ssb3. Similarly, in the presence of genotoxins, deleting Ssb3 appeared to only slightly enhance the growth defect of *mus81Δ* cells, indicating a close functional relationship between Ssb3 and Mus81 (Figure 9B).

Our studies identified a negative genetic interaction between *ssb3Δ* and *rhp55Δ*, with the double mutant being similar to the *rhp51Δ* strain (Figure 8A). Rhp55-Rhp57 protein complex is one of two Rhp51 mediators, with the other consisting of Sfr1 and Swi5, which form a protein complex that serves as an alternative mediator for Rhp51 [43], [44]. Inactivating both mediators severely impairs Rhp51 function. In view of these relationships, we tested the genetic interactions between *ssb3Δ* and *sfr1Δ* or *swi5Δ*. In contrast to the interaction between *ssb3Δ* and *rhp55Δ* in the absence of genotoxins (Figure 8A), our data indicated there was little or no interaction between *ssb3Δ* and *sfr1Δ* or *swi5Δ* in the absence of genotoxins (Figure 9C). However, there were obvious negative interactions between *ssb3Δ* and *sfr1Δ* or *swi5Δ* in the presence of MMS or CPT, and weaker interaction in double mutant cells treated with UV or HU (Figure 9C). The possible interpretations of these data are discussed below.

## Discussion

As a central component of all DNA transactions involving ssDNA, RPA has been the subject of many genetic, biochemical and structural studies. The large majority of the *in vivo* functional studies have been carried out with *S. cerevisiae*, in which it is clearly established that all 3 subunits of RPA are essential for cell viability. It was reasonable to assume the same was true in all organisms. We were therefore surprised to find that the small subunit of RPA was apparently not essential in fission yeast. To eliminate doubts, we reengineered an *ssb3Δ* mutation to completely eliminate the *ssb3* ^+^ open reading frame, and found that this mutant was also viable. From these studies we conclude that a heterodimeric complex consisting of Rad11^Rpa1^and Ssb2^Rpa2^ can carry out the essential DNA replication functions of RPA in fission yeast. As functional studies unfold in other model organisms, it will be interesting to determine whether the essentiality of Rpa3 is the rule or the exception.

Whilst our studies demonstrate that Ssb3 is not required for the essential functions of RPA, they nevertheless show that Ssb3 has important effects on RPA function. One observation supporting this conclusion is the temperature sensitive genetic interaction between Rad11-GFP and *ssb3Δ*. It is likely that both alleles modestly destabilize or impair the function of RPA complex, to the degree that combining the alleles causes an acute temperature sensitive phenotype. This hypothesis is consistent with data indicating that Rpa3 mediates protein interactions that help to stabilize the RPA heterotrimer [12], [13].

In the absence of exogenous DNA damaging agents, *ssb3Δ* mutants have a modest growth defect, moderate cell elongation dependent on Chk1, and an elevated number of Rad11^Rpa1^ and Rad22^Rad52^ foci. These phenotypes most likely result from defects in DNA replication, leading to DNA structures recognized as DNA lesions by the DNA damage checkpoint and HR repair machinery. However, *ssb3Δ* and *chk1Δ* mutations do not have an obvious synergistic growth defect. These data suggest that the DNA lesions leading to a Chk1-dependent mitotic delay in *ssb3Δ* cells are unlikely to be DSBs. In support of this conclusion, we did not detect a obvious genetic interaction between *ssb3Δ* and *rhp51Δ* when mutants were tested in the absence of genotoxins. Thus, it is likely that *ssb3Δ* cells accumulate gapped ssDNA structures that activate the DNA damage checkpoint and are substrates for Rad22.

We found that *cds1Δ* partially suppresses the MMS and CPT sensitivity of *ssb3Δ* cells (Figure 3D). This genetic interaction was unexpected. The toxicity of MMS and CPT is thought to derive primarily from replication fork collapse; therefore, these data suggest that activation of the replication checkpoint is detrimental to restoration of collapsed replication forks in *ssb3Δ* cells. It will be interesting to discover which substrates of Cds1 kinase mediate this effect.

Whilst *ssb3Δ* cells are sensitive to CPT, MMS and UV, they display only weak sensitivity to IR. The toxic effects of CPT, MMS and UV are thought to arise primarily from creating DNA lesions that interfere with DNA replication, whereas IR directly causes DSBs in all phases of the cell cycle. These data suggest that Ssb3 is not critical for typical HR-mediated DSB repair in mitotic cells, which occurs through a synthesis-dependent strand-annealing (SDSA) pathway requiring Rad22^Rad52^, Rhp51^Rad51^, and other core HR proteins. Furthermore, *ssb3Δ* cells exhibit only a very minor defect in spore viability. Since formation of viable spores depends on carrying out HR repair of programmed meiotic DSBs through a double-strand break repair (DSBR) pathway requiring resolution of Holliday junctions by Mus81-Eme1 resolvase [21], our findings show that both pathways of HR repair are largely functional in the absence of Ssb3. However, these data do not exclude the possibility that deletion of *ssb3* ^+^ might have modest effects in meiotic recombination.

The HR-mediated repair of CPT-induced broken replication forks also depends on Rad22, Rhp51 and Mus81, and our data indicate that Ssb3 plays a relatively important role in this pathway. It is unclear why Ssb3 should appear to be more important for repair of CPT-induced damage than IR-induced DSBs. Ssb3 might have an important role in stabilizing stalled replication forks, such as those that are proposed to form as the result of positive supercoils forming ahead of the fork in cells treated with CPT [19]. It is noteworthy that previous genetic studies have identified other genome maintenance factors that are not required for repair of IR-induced DSBs but are critical for survival of CPT treatment. The most relevant factors may be Mus81-Eme1 and Mms1-Mms22 protein complexes, which are critical for survival of CPT treatment but are not required for repair of IR-induced DSBs [21]-[23], [25], [28]. In this respect, it is likely to be particularly significant that *ssb3Δ* displays little or no synergistic genetic interactions with *mus81Δ*, *mms1Δ* and *mms22Δ* mutations in the absence or presence of genotoxins, indicating that there are likely to be close functional connections between the Ssb3-dependent functions of RPA and the activities of Mus81-Eme1 Holliday junction resolvase and Mms1-Mms22 protein complex. This possibility is entirely consistent with studies showing that *mms1Δ* and *mms22Δ* have negative genetic interactions with mutations of many genome maintenance genes, but not with *mus81Δ* [24], [25], [28]. However, it is important to note that unlike Mus81, Ssb3 is not required for resolution of Holliday junctions in meiosis, and thus it is unlikely that Ssb3 has an integral role in the Mus81-dependent resolution of Holliday junctions that form upon restoration of collapsed replication forks [21], [44].

Regardless of the genotoxins assayed in our studies, the phenotypes of *ssb3Δ* cells do not match that of an *rhp51Δ* mutant that is severely defective in HR-mediated DSB repair. However, the phenotypes of *ssb3Δ* and *rhp55Δ* mutants are similar, and there is a strong genetic interaction when *ssb3Δ* and *rhp55Δ* are combined. In fact, the *ssb3Δ rhp55Δ* double mutant is approximately equivalent to *rhp51Δ*. These data suggest that there is a nearly complete breakdown of HR in the *ssb3Δ rhp55Δ* double mutant. Rhp55 is a Rad51 paralog that forms a heterodimer with Rhp57. Studies of the analogous Rad55-Rad57 complex in budding yeast have shown that it can function as a mediator in the strand-exchange reaction necessary for Rad51 to nucleate on ssDNA in the presence of RPA [45]. One possible interpretation of these findings is that both *ssb3Δ* and *rhp55Δ* mutations cause defects in Rad51 nucleation on ssDNA, resulting in a synergistic interaction of the mutations. RPA foci are actually enhanced in *ssb3Δ* cells, suggesting that a key role of Ssb3 may be promoting the disassembly of RPA from ssDNA.

Interestingly, in the absence of exogenous genotoxins, *ssb3Δ* does not have obvious genetic interactions with mutations deleting genes encoding Sfr1 or Swi5, which form an alternative mediator complex for Rhp51 [46], [47]. These data are consistent with the possibility that Ssb3 might act with Sfr1-Swi5 complex. However, when tested in the presence of genotoxins, there are clear negative genetic interactions involving *ssb3Δ* and *sfr1Δ* or *swi5Δ*. The same is true for *ssb3Δ* and *rhp51Δ* mutations. Thus, Ssb3 might act with the Sfr1-Swi5 complex, but it also has functions that do not involve Rhp51 and its mediators.

## Materials and Methods

### Strains and genetic methods

The strains used in this study are listed in Table S1. Standard procedures and media for *S. pombe* genetic and biochemical analyses were used as previously described [48]. Cells were cultured in YES (yeast extract, glucose and supplements) or EMM (defined minimal medium with supplements) as described [48]. The complete deletion of the *ssb3* open reading frame was made using pFA6a-KanMX6 as template and the primers “Ssb3 KanMX6 forward” (5′-TCG TGT CAA CAA GTA GTT AAC TAC CTG GTC TGA TAC ATA CTT CAC TTC CAC CAC TTT ATA AAC AAC GCG TAT AAA ATA ATC GGA TCC CCG GGT TAA TTA A-3′) and “Ssb3 KanMX6 reverse” (5′-CGT TTA TTC TTC CAT GTT TAT TTG TAC TGT GCA TGA GAA ATG AAA GAG AAA TCT GTG TTG TAT GAT CCA TAA AAT GTT TCG AAT TCG AGC TCG TTT AAA C-3′). The PCR product was transformed into PR110 (*h* ^+^ *leu1-32 ura4-D18*) cells by electroporation, G418-resistant colonies were obtained, and PCR and DNA sequencing verified *ssb3* disruption.

### Microscopy

Cells were photographed using a Nikon Eclipse E800 microscope equipped with a Photometrics Quantix CCD camera and IPlab Spectrum software. All fusion proteins were expressed at their own genomic locus. Rad22-YFP and Ssb3-GFP/Rad22-RFP-expressing strains were cultured in EMM, while Ssb3-GFP and Rad11-GFP-expressing strains were grown in YES until mid-log phase for foci quantification assays. Experiments with Rad11-GFP expressing strains were carried out at 25°C because of the thermosensitivity of the *rad11-GFP ssb3Δ* strain. In the case of CPT treatment, 30µM CPT in DMSO (or DMSO alone as a control, 0.3% final concentration) was added to mid-log phase cultures for 4 hrs at 30°C, washed out and cells were resuspended in YES for foci quantification. In all cases, at least 800 nuclei were scored in three independent experiments. All microscopy was conducted with live cells. Cell length, nuclei number and position, and the presence of a division plate were used to assess cell cycle position.

### Survival assays

In chronic exposures to drugs, mid-log phase cultures were resuspended to 0.5 OD600 and serially diluted tenfold. Dilutions were spotted onto YES agar plates containing the indicated amounts of CPT, MMS or HU. Note that the effective concentration of these drugs, particularly MMS, can vary depending on the age and source of the stock solution, which accounts for the different concentrations used in some of the survival assays. For exposure to IR, cells were irradiated using a ^137^Cs source with the indicated dose and then serially diluted onto triplicate YES plates. In the case of UV treatment, cells were serially diluted onto YES plates and irradiated using a Stratagene Stratalinker UV source. Cell survival was determined after 3–4 days at 30°C unless otherwise indicated.

### Immunoblotting

Whole cell extracts were prepared from exponentially growing cells disrupted in lysis buffer (50mM Tris-HCl pH 8, 150mM NaCl, 2.5mM EDTA, 10% Glycerol, 0.2% NP-40, 50mM NaF, 5mM PMSF, and Complete Protease Inhibitor tablets (Roche)) with a glass bead beater and resolved in 8% SDS-PAGE. Proteins were transferred to nitrocellulose membranes, blocked with 5% dry milk, 0.05% Tween-20 in TBS (150mM NaCl, 50mM Tris-HCl pH 7.6) and probed with HA (Roche) antibody.

## Supporting Information

Table S1 Strains used in this study.
(0.09 MB DOC)
