# Supplementary material for: Critical Functions of Rpa3/Ssb3 in S-Phase DNA Damage Responses in Fission Yeast
Source: PLoS Genet. 2010 Sep 23;6(9):e1001138. doi: 10.1371/journal.pgen.1001138 (PMC2944793; doi:10.1371/journal.pgen.1001138)
Supplement: Table S1 — Strains used in this study. (0.09 MB DOC) [file pgen.1001138.s001.doc]

**Table S1. Fission yeast strains use in this study.** All strains are *leu1-32 ura4D-18* unless otherwise indicated.

| Strain | Genotype |
| --- | --- |
| PR37 | *h^–^ leu1^+^ ura4^+^* |
| PR110 | *h^+^* |
| SC4461 | *h^-^ leu1^+^ ura4^+^ ssb3::kanMX4* |
| SC4460 | *h^+^ ssb3::kanMX4* |
| SC4462 | *h^+^ ssb3::kanMX6* |
| SC4467 | *h^+^ ssb3-GFP:kanMX6* |
| EN3239 | *h^+^ ade6-M210 rad11A* |
| SC4501 | *h^+^ ade6-M210 rad11-D233Y* |
| EM4889 | *h^+^ rad11-GFP::hphMX6* |
| SS3067 | *h^-^ chk1::ura4^+^* |
| KT2751 | *h^-^ cds1::ura4^+^* |
| BF2479 | *h^+^ rad3::ura4 =^+^* |
| LLD3427 | *h^-^ chk1-9myc2HA3His:ura4^+^* |
| SC4474 | *h^+^ ssb3::kanMX4 chk1::ura4^+^* |
| SC4473 | *h^-^ ssb3::kanMX4 cds1::ura4^+^* |
| SC4475 | *h? ssb3::kanMX4 rad3::ura4^+^* |
| SC4476 | *h^+^ ssb3::kanMX4 chk1-9myc2HA3His:ura4^+^* |
| EN3222 | *h^-^ rad22-YFP:kanMX6* |
| SC4477 | *h^-^ ssb3::kanMX4 rad22-YFP:kanMX6* |
| SC4478 | *h? ssb3-GFP:kanMX6 rad22-RFP:hphMX6* |
| EN3182 | *h^-^ swi1::KanMX* |
| EN3366 | *h^-^ swi3::KanMX* |
| SC4485 | *h^+^ ssb3::kanMX4 swi1::kanMX* |
| SC4486 | *h^-^ ssb3::kanMX4 swi3::kanMX* |
| PS2594 | *h^+^ his7-? rfc3-1* |
| EM4890 | *h^-^ ctf18::kanMX6* |
| SC4497 | *h^-^ his7-? ssb3::kanMX4 rfc3-1* |
| SC4496 | *h^+^ ssb3::kanMX4 ctf18::kanMX6* |
| NR1587 | *h^-^ ade6-704 rad13::ura4^+^* |
| PS2443 | *h^-^ leu1^+^ swi10:: ura4^+^* |
| PS3126 | *h^-^ ade6-704 rad2::ura4^+^* |
| SC4487 | *h^-^ ssb3::kanMX4 rad13::ura4^+^* |
| SC4488 | *h^+^ ssb3::kanMX4 swi10::ura4^+^* |
| SC4491 | *h^-^ ssb3::kanMX4 rad2::ura4^+^* |
| TMN3319 | *h^+^ ade6-M216 his3-D1 rhp51::ura4^+^* |
| PS2386 | *h^+^ ade7-152 rhp55::ura4^+^* |
| SC4482 | *h^+^ ssb3::kanMX4 rhp51::ura4^+^* |
| SC4483 | *h^+^ ssb3::kanMX4 rhp55::ura4^+^* |
| CD4089 | *h^+^ mms22::hphMX6* |
| EN3169 | *h^-^ brc1::kanMX6* |
| SC4494 | *h^-^ ssb3::kanMX4 mms22::hphMX6* |
| SC4495 | *h^+^ ssb3::kanMX4 brc1::kanMX6* |
| SC4479 | *h? ssb3::kanMX4 rad11-GFP:hphMX6* |
| CD4682 | *h^+^ mms1::hphMX6* |
| CD4692 | *h^+^ mus81::natMX6* |
| OL4943 | *h^+^ ssb3::kanMX6 mms1::hphMX6* |
| OL4944 | *h^+^ ssb3::kanMX6 mus81::natMX6* |
| OL4945 | *h^+^ swi5::kanMX6* |
| OL4946 | *h^-^ sfr1::kanMX6* |
| OL4947 | *h^+^ swi5::kanMX6 ssb3::kanMX6* |
| OL4948 | *h^+^* *sfr1::kanMX6 ssb3::kanMX6* |
| SC4499 | *h^+^ ssb3-RFP:kanMX6 rad11-GFP:hphMX6* |
